# The geography of maternal and newborn health: the state of the art

**DOI:** 10.1186/s12942-015-0012-x

**Published:** 2015-05-27

**Authors:** Steeve Ebener, Maria Guerra-Arias, James Campbell, Andrew J. Tatem, Allisyn C. Moran, Fiifi Amoako Johnson, Helga Fogstad, Karin Stenberg, Sarah Neal, Patricia Bailey, Reid Porter, Zoe Matthews

**Affiliations:** Gaia GeoSystems, Muscat, Oman; ICS Integrare, Barcelona, Spain; WHO, Geneva, Switzerland; WorldPop, University of Southampton, Southampton, UK; USAID, Washington, DC USA; University of Southampton, Southampton, UK; NORAD, Oslo, Norway; AMDD and FHI 360, Durham, USA; The University of Texas at Austin, Austin, USA

**Keywords:** Maternal health, Newborn health, Geography, GIS, Inequalities, Millennium development goals

## Abstract

As the deadline for the millennium development goals approaches, it has become clear that the goals linked to maternal and newborn health are the least likely to be achieved by 2015. It is therefore critical to ensure that all possible data, tools and methods are fully exploited to help address this gap. Among the methods that are under-used, mapping has always represented a powerful way to ‘tell the story’ of a health problem in an easily understood way. In addition to this, the advanced analytical methods and models now being embedded into Geographic Information Systems allow a more in-depth analysis of the causes behind adverse maternal and newborn health (MNH) outcomes. This paper examines the current state of the art in mapping the geography of MNH as a starting point to unleashing the potential of these under-used approaches. Using a rapid literature review and the description of the work currently in progress, this paper allows the identification of methods in use and describes a framework for methodological approaches to inform improved decision-making. The paper is aimed at health metrics and geography of health specialists, the MNH community, as well as policy-makers in developing countries and international donor agencies.

## Introduction

Maternal and newborn health (MNH) care refers to ‘those activities whose primary purpose is to restore, improve and maintain the health of women and their newborn during pregnancy, childbirth and the 7-day postnatal period’ 1[1]. World Health Organization (WHO) guidance on the activities that should be included in MNH care includes the essential packages of interventions that address pregnancy, childbirth, postpartum care to mothers and care to newborns [2]. MNH outcomes are subject to wide geographic inequities, both at the regional, national and sub-national levels. The location of services is a key determinant of women’s access to MNH care [3–6], thus the application of geographic approaches and geographic information systems (GIS) to MNH is critical to assess the situation and needs and guide policy decisions, leading to improved services and care and thus to improved MNH outcomes [7, 8].

The health and survival of women and their newborn babies has been a key priority in public health for decades, as highlighted by two of the eight ‘Millennium Development Goals’ (MDGs), from the Millennium Declaration endorsed by 189 member states in the year 2000 [9]. Goal number five of these international priorities aims to cut maternal mortality by three-quarters from 1990 to 2015 and goal four focuses on reducing newborn and child mortality by two-thirds. However, as the deadline for these goals approaches, it has become clear that of the eight original goals (which also target poverty, malnutrition, educational levels, environmental conditions and sanitation) goals four and five are the least likely to be achieved in the time span set. Women still die in childbirth—289 000 per year in 2013 [10]—especially in low income countries and although child mortality is dropping, neonatal deaths have not decreased at the same rate, now representing 44 % of all deaths under 5 years [11].

In recognition of poor progress and a lack of information, the UN Secretary-General commissioned a new Global Strategy for Women’s and Children’s Health in 2010 [12] to accelerate progress in reducing maternal and newborn mortality ahead of the rapidly approaching MDG deadline. As a result of this, a Commission on Information and Accountability for Women’s and Children’s Health was established [13] to systematically track progress as well as collect health system data that support the survival and wellbeing of pregnant women and their newborns. These data include indicators that measure the coverage of MNH services, including antenatal care and skilled birth attendance. 2 Availability of these services is an important step to tackling the problem, but services also need to be accessible to women, acceptable to them and their families and crucially, of a sufficient quality. Finally, women must be able and enabled to make the decision to seek care, otherwise the availability, accessibility, acceptability and quality of services becomes irrelevant [14, 15].

Thus far, the measurement of progress has focused on aggregate indicators at the country level, but increasingly there are concerns that even where progress has been made, it is subject to wide inequalities [16]. Even in countries where national maternal and newborn mortality rates have declined, there may be subgroups where survival rates and access to services have not changed or have worsened. Investigation of sub-national situations through a geographical analysis is now increasingly required.

This paper arises from a workshop held in March 2013 entitled ‘Geography 4 MNH’^3^, to explore and describe the current “State of the Art” of MNH mapping. The workshop, hosted by Instituto de Cooperación Social Integrare and the University of Southampton, enabled collaboration across research and development agencies to identify existing published and grey literature, describe current work in this field and develop a framework to accelerate the use of this capacity in priority countries with high maternal and neonatal mortality, in order to improve MNH outcomes.

The urgency with which the MNH community must embrace the geographical interpretation of existing databases is particularly critical, both in advance of the endpoint for the MDGs and to inform the post-2015 development agenda for health and the new generation of goals and information requirements over the period 2015–2035 [17]. Successful examples of the application of geographic approaches and use of geospatial data to improve MNH services show the potential of these technologies when integrated within national health strategies. Examples include the use in Bangladesh of an interactive EmONC monitoring GIS system that tracks quality and gaps in services [7]; the use of GIS to evaluate the impact of a community-based health initiative in rural Ghana [8]; and the UK’s teenage pregnancy reduction strategy, which used ward level data of conceptions in young women under 18 years to assist in targeting interventions where most needed [18].

## Review

### What has been done so far – a rapid review of previous studies

A rapid review was conducted of the published and grey literature on geographical approaches related to the health and survival of women and children. To identify key sources, an exercise was conducted with technical and academic experts, who were invited to contribute relevant studies to be reviewed. Subsequently, a “snowballing technique” was used to identify further valuable references from these initial sources and so on. The selection was based on a review of title, abstract and key words related to geography and MNH, including: distance, emergency obstetric care, geographic information systems, health facilities, health services accessibility, maternal health, maternal health services, newborn, pregnancy, spatial analysis, *etc.* The start date for inclusion was the year 2000. As well as the articles identified through the “snowballing technique”, additional studies were identified independently by the authors and added to the review.

In total, 33 studies were identified with a focus on MNH. Studies that focus on particular health problems appear to be less common than those that focus on overall disease surveillance, access or risk analysis. A systematic review in 2011 found 621 articles relating to the use GIS in health, out of which 227 related to disease surveillance, 189 to risk analysis (usually linked to environmental health), 138 to health access and planning, 115 on community profiling and 17 on general or methodological issues [19].

An extraction table was used to compile key information from each of the 33 retrieved papers, namely: year of publication, region and country of implementation, geographical scope, objectives and themes covered, major findings, geographical data layers included, other data collected and GIS approaches used. Table 1 presents selected dimensions of the literature retrieved.

**Table 1:** Selected information from a rapid literature review on mapping for MNH

Publication	Region	Scope	Theme	GIS approaches used
Sudhof *et al.*, 2013 [39]	Africa	Sub-district	Geographic access to MNH care	Spatial modelling (travel time to facilities along road networks)
Bowie, 2013 [40]	Africa	National	Geographic access to MNH care	Spatial modelling (travel time to facilities)
Ismaeel & Jabar, 2013 [41]	Middle East	City	Support system for MNH care	Spatial analysis (straight-line distance to facilities)
Echoka *et al.*, 2013 [42]	Africa	Sub-national	Geographic access to MNH care	Spatial analysis (straight-line distance to facilities)
Lohela *et al.*, 2012 [43]	Africa	National (rural)	Impact of geographical access on newborn mortality	Spatial analysis (straight-line distance to facilities)
Gething *et al.*, 2012 [44]	Africa	National	Geographic access to MNH care	Spatial modelling (travel time to facilities along road networks)
Kyei *et al.*, 2012 [45]	Africa	National (rural)	Impact of geographic access on ANC utilization	Spatial analysis (straight-line distance to facilities)
Pilkington *et al.*, 2012 [46]	Europe	National	Impact of geographical access on delivery	Spatial analysis (distance to facilities along road networks)
Bailey *et al.*, 2011 [47]	Africa	Sub-national	Geographic access to MNH care	Spatial modelling (travel time to facilities along road networks)
Gjesfjeld & Jung, 2011 [48]	North America	Sub-national	Geographic access to MNH care	Spatial analysis (straight-line distance to facilities)
Fisher & Myers, 2011 [49]	Asia	Sub-national	Geographic access to MNH care	Spatial modelling (travel time to facilities along road networks)
Gabrysch *et al.*, 2011 [50]	Africa	National (rural)	Impact of geographical access on use of services.	Spatial analysis (straight-line distance to facilities)
Cordivano, 2011 [51]	North America	City	Impact on maternity ward closure	Spatial analysis (straight-line distance to facilities, analysis of facility based buffers to determine catchment areas)
Redshaw *et al.*, 2011 [52]	Europe	National	Maternity care organization	Thematic mapping (density of facilities)
Massey, 2011 [53]	Africa	National	Geographic patterns of MNH	Thematic mapping (analysis of hot-spots of adverse outcomes)
Simoes & Almeida, 2011 [54]	Latin America	City	Impact of geographic access on MMR.	Spatial analysis (distance to facilities along road networks)
Zahinos Ruiz, 2010 [55]	Africa	Sub-national	Assess effectiveness (MWHs)	Spatial analysis (investigation of facility based buffers to determine catchment areas)
Saugene, 2010 [56]	Africa	Sub-national	Planning of ambulance routes	Spatial modelling (travel time to facilities along road networks)
Malqvist *et al.*, 2010 [57]	Asia	Sub-national	Impact of geographic access on mortality and care utilization.	Spatial analysis (straight-line distance to facilities)
Pilkington *et al.*, 2008 [58]	Europe	National	Impact on maternity unit closure	Spatial analysis (straight-line distance to facilities and, analysis of facility based buffers to determine catchment areas)
Dummer & Parker, 2004 [59]	Europe	Sub-national	Accessibility and infant death risk	Spatial modelling (travel time to facilities along road networks)
Balk *et al.*, 2003 [20]	Africa	Region	Impact of geography on child/infant mortality	Spatial analysis (straight-line distance to facilities and analysis of facility based buffers to determine catchment areas)
Ayeni, 2002 [60]	Africa	City	Birth weight analysis	Thematic mapping (overlaying different map layers and small area analysis)
Leewannapasai *et al.*, 2001 [61]	Asia	Sub-district	Geographic access	Thematic mapping (overlaying different map layers)
Jain *et al.*, 2015 [62]	Asia	Sub-national	Impact of geographical access on institutional deliveries	Spatial analysis (distance to facilities along road networks)
Tatem *et al.*, 2014 [63]	Asia and Africa	National	Geographic distribution of women of child-bearing age, pregnancies, births and facilities	Thematic mapping (overlaying different map layers)
Heard *et al.*, 2004 [64]	Africa	National	Impact of geographic access on service utilization	Spatial analysis (straight-line distances to facilities)
Blanford *et al.*, 2012 [65]	Africa	National	Geographic access	Spatial modelling (travel time to facilities along road networks)
Nesbitt *et al.*, 2014 [66]	Africa	Sub-national	Accuracy of different measures of geographic access as proxies for access to services.	Spatial analysis (straight-line distance to facilities, distance to facilities along road network) and spatial modelling (travel time to facilities along road networks).
Chong *et al.*, 2013 [67]	Oceania	City (sub-region)	Use of geographic methods to identify high-risk communities	Thematic mapping (spatial clusters of at risk patients)
Amoako Johnson *et al.*, 2015 [8]	Africa	National (rural)	Impact of geographical access on use of skilled birth attendance.	Spatial analysis (distance to facilities along road networks)
Ravelli *et al.*, 2010 [4]	Europe	National	Impact of geographical access on neonatal outcomes.	Spatial modelling (travel time to facilities along road networks)
Okwaraji *et al.*, 2012 [3]	Africa	Sub-national	Impact of geographical access on under 5 mortality	Spatial modelling (travel time to facilities)

This review shows a clear increase in the number of GIS for MNH publications since 2010. The studies cover most regions of the world. As expected, in view of its high maternal and infant mortality levels [10] Africa has received the greatest research focus with 19 studies. Asia (including South, South-East and East Asia) and Europe follow with 5 studies each.

The majority of the studies conduct their analysis at the national or sub-national level. One of the studies [20] covers the broader geographical region of West Africa, but individual analyses are conducted at the level of each country in the region.

The most common study objective is to examine geographic access to health services. 4 A variety of different GIS techniques are used to map, extract or model MNH related indicators, from simple thematic mapping, spatial analysis (straight line distance, buffers) or spatial modelling involving travel time cost surfaces, which take transport networks and other geographical features into account.

### Work in progress

One of the key purposes of convening parties actively mapping the geography of MNH was to identify the work in progress that may influence the ‘state of the art’. Table 2 provides examples of on-going work, looking at the who?, what? and where? for each project (full descriptions available on request).

**Table 2:** Examples of work in progress on mapping for MNH

Who?	What		Where?
Saving Mothers Giving Life (SMGL)/ Centers for Disease Control (CDC)	Thematic Mapping	Maps on maternal mortality and access to facilities	Uganda
Evidence for Action (E4A) [68]/ White Ribbon Alliance (WRA) [69]	Thematic Mapping	Thematic mapping of selected indicator at the sub national level (2013)	Ethiopia
		Atlas of Birth, extensive mapping.	Ghana
Countdown to 2015 [70]	Thematic Mapping	Country case study – focus on maternal mortality reduction	Bangladesh
United Nations Children’s Fund (UNICEF)	Thematic Mapping	Sharpening Maternal, Newborn and Child Health (MNCH) plans. Using Dev Info maps for program planning	Senegal
USAID	Thematic Mapping, Spatial analysis and modeling	Emergency Obstetric and Newborn Care (EmONC) availability and accessibility coverage (In collaboration with FHI 360, MEASURE Evaluation and AMDD)	Mozambique and Ethiopia
USAID	Spatial analysis	MNH Mapping Modelling sub-national estimates from 2010 Bangladesh Maternal Mortality Survey; Other work under development	Bangladesh
Health 4+ (H4+)/ Technical Working Group and Secretariat + Muskoka [71]	Spatial analysis and modelling	High Burden Countries Initiative (HBCI) Midwifery Workforce Assessment (2012–13). Determining sub-national population needs (through a proxy of the estimated number of pregnancies) in relation to the supply of EmOC facilities and the MNH workforce.	Afghanistan, Bangladesh, Benin, Democratic Republic of Congo, Ethiopia, Ghana, Guinea, India (2 States), Mozambique (in collaboration with USAID), Nigeria, Tanzania, Togo
USAID	Spatial analysis and modelling	Analysis of EmOC availability and accessibility coverage; feasibility of real time mapping.	Malawi
Southampton University	Spatial analysis and modelling	EmOC availability and accessibility coverage.	Ghana
World Health Organization (WHO)	Spatial analysis and modelling	EmOC availability and accessibility coverage as well as scaling up costing analysis	Burkina Faso, Cambodia, Laos, Malawi, Philippines

The work in progress is consistent with the findings of the literature review, both in terms of countries studied and methods employed. Several partners are involved in these activities, creating some overlap in the countries being covered and calling for coordination.

These different projects span a wide range of methods: from the use of thematic maps to visualize indicators at different levels of disaggregation, to the use of complex spatial analysis and modeling techniques in order to assess the accessibility of MNH services. In each case, the power of GIS is used to represent, extract or produce new evidence of interest to the decision-making process (see the next section for a description of the benefits of each method).

Of particular interest is that several of the multi-country initiatives (H4+, USAID and WHO) have independently adopted the Tanahashi framework of effective coverage [14] to guide their technical analyses. This framework considers the dimensions of availability, accessibility, acceptability and quality of services when measuring population and facilitates the identification of bottlenecks and solutions, as appropriate to the geography. This convergence between geography and therefore indirectly GIS and the Tanahashi framework is encouraging and provides scope for ‘good practise’ to be documented and disseminated. Another encouraging finding is that professional learning and capacity-building are incorporated within some of the activities with a longer duration, *e.g.* the multi-country work supported by WHO and Evidence for Action (E4A). Knowledge transfer on the methods, analysis and policy implications will be critical to integrate the geography of MNH into national policy and planning cycles in the future.

The field of Small Area Estimation (SAE) is also emerging in the geographical analysis of MNH and allows the estimation of measures for very small geographies by linking the variable of interest with covariate or contextual information from census, administrative or geographic information systems data to derive model-based estimates for small areas. If data on the variable of interest are available only for a limited number of individuals in the small area, the covariate information is available for all individuals in the area and is assumed to explain part of the between area variability [21, 22]. Two broad types of small-area models exist. Area-level models are used when the auxiliary information is available only at the area level [22] and nested error unit-level models are used the auxiliary information is available at the individual level and is related to the variables of interest at the unit-level [23]. Such methods have been employed widely in high income countries and for economic variables such as poverty indices [24, 25] – but less so for health variables [26, 27]. Amoako-Johnson *et al.* [26] apply SAE for skilled attendance and unmet need for contraception and Ahmed *et al.* [28] have reviewed maternal mortality ratios in Bangladesh.

In demographic and health research, particularly in the estimation of maternal and newborn healthcare indicators there are methodological as well as data challenges that limit the application of small area estimation techniques [29–32]. Despite these limitations, more and more low and middle income countries are using this type of analysis to reduce the extent of inequality in access to health care as, in these countries, local level statistics on healthcare provision and outcomes are rare if not non-existent for aid planning, monitoring and evaluation of programs.

### Moving towards comprehensive GIS for MNH

The review of the literature and work in progress presented here has identified three types of GIS methods, namely:

1. Thematic mapping (creation of maps to convey information about a topic or theme);
2. Spatial analyses (extraction or creation of new information from spatial data);
3. Spatial modelling (spatial analysis that includes the use of a mathematical model to simulate natural or anthropogenic phenomena).

Each of these methods adds specific value to the policy discussion. Thematic maps are powerful instruments that allow visualizing sub national information from a geographic perspective. Spatial analysis techniques, including spatial modelling, are advanced methods that are used to provide a more in-depth analysis and understanding of the health systems factors and behaviours behind MNH related health outcomes.

These methods are listed not only in order of increasing complexity but also by their reliance on data in terms of volume, completeness (having all the data on the map), timeliness (chronologic consistency between the different sources of data) and accuracy (precise location of each geographic object), and the advanced level of GIS skills required to apply them.

The possibility for countries to benefit from any of these applications will be influenced by the availability and quality of data and technical resources. Thus, an appropriate and adequately financed institutional framework is needed, to sustain and improve the availability and quality of the data and to further enhance the required technical skills and equipment. Table 3, while not exhaustive, provides an overview of the components that would support such an institutional framework to advance the application and implementation of GIS in MNH.

**Table 3:** Required data, data quality issues and required GIS skills, by GIS approach to mapping for MNH

	Required data	Important GIS data quality issues to be addressed	Required GIS skills
Thematic mapping	Statistical data (indicators) and GIS data containing the extension of the geographic objects to which the statistical data are attached	Completeness and timeliness	Basic GIS training (Knowledge for producing relevant thematic maps)
Spatial analysis	Location of the different geographic objects necessary to create or extract the new information (location of the households, health facilities,…)	Completeness, timeliness and accuracy	Intermediate GIS training (GIS data management, spatial analysis functions)
Spatial modelling (geographic access)	Location of the health facilities providing the concerned MNH care; Spatial distribution of the population in demand (pregnant women, births,…); Road network; hydrographic network; Digital Elevation Model (DEM); Land cover and coverage capacity of each facility if human resources taken into account in the analysis		Advanced GIS training (GIS data management and manipulation, use of advanced GIS functions)

Additional considerations from the authors’ experience to complement this table include:

1. The lowest available level of disaggregation should be used to avoid masking potential pockets of heterogeneity in aggregate data;
2. Finding accurate GIS data is often a challenge as indicators might have been collected according to different types of divisions (administrative, statistical or even health) and/or at different periods in time;
3. A particular challenge resides in the use of MNH indicators that would be available at the health facility level (presence of EmONC signal functions, for example). A master list containing all national health facilities (rather than a sample) as well as their geographic location (latitude/longitude) is an absolute necessity. Ensuring the use of such registry as well as the integration of geography in any health facility based survey would also contribute to facilitating the use of GIS as well as the comparison between maternal/neonatal health services provision and outcomes [33];
4. At all levels it is important that the GIS technician works as part of a team of experts to produce timely, reliable and relevant results for decision-making.

Table 3 emphasises the importance of having a strong health information system in place but also for this system to integrate and contextualise both geography and time effectively and accurately. The former requires complete and up-to-date registries, to be established and maintained for each of the geographic objects to which MNH related statistics can be attached (administrative, statistical, health divisions, health facilities). In addition, advanced techniques such as spatial analysis or spatial modelling require the use of GIS data under the mandate of institutions outside of the health sector (for example, National Statistical Office, National Mapping Agency and/or National Road Authority). All these institutions therefore need to use compatible data based on common standards.

The use of GIS also requires that the health sector has the necessary capacity (human resources, software and hardware) in place. While this capacity is increasing, thanks partly to the availability of open source software, in a large number of Ministries of Health it remains at a basic level and limits analysis to thematic mapping. Sustained implementation and continued expertise in countries are needed. Good news on this comes from non-health sectors such as agriculture – where GIS expertise is being utilized more and more frequently and from Health Management Information Systems development – where growing IT teams are increasingly embedding GIS within the standard monitoring tools. The need to support and harness GIS systems and expertise is also explicit in post −2015 plans for statistical development in countries – for example in the forthcoming ‘road map for health measurement’ to be discussed at a summit in Washington June 2015 [34], and among the new leaders of the Inter Agency Group for the SDGs [35], which is being led by national statistical agencies and whose needs for capacity development are increasingly being heard.

Despite these challenges the emphasis on reducing maternal and newborn mortality is an opportunity for new synergies. The health sector can use MNH as the driver to improve GIS capacity, thus benefiting all health initiatives. This win-win situation would be possible through an institutional framework in which:

1. The health sector is actively involved in discussions aimed at standardising their own geospatial information. The forum for this is generally referred to as the National Spatial Data Infrastructure (NSDI);
2. International health partners supporting MNH program activities strengthen the existing GIS capacity through a coordinated approach; independently from specific health program support;
3. Institutions involved in the strengthening of health information systems include the integration of geographic and time dimensions in their approach;
4. MNH oriented programs systematically consider geographic analysis and GIS as a tool to improve the implementation and monitoring of MNH programs.

The institutional framework would ideally be developed and taken forward as an integral component of the UN Secretary-General’s Every Woman, Every Child initiative, with linkages to the Commission on Information and Accountability for Women’s and Children’s Health (CoIA) and the independent Expert Review Group (iERG).

## Conclusions

Despite an encouraging trend of increased interest in applying GIS technology for improving MNH outcomes in recent years, much remains to be done to develop national capacity in low- and middle-income settings, to perform geographical analysis.

This state of the art serves as a baseline to raise awareness of the potential that GIS methods have to offer to MNH and provides the components of an institutional framework that we recommend be included in the further work of the CoIA and the iERG. It calls for better communication and coordination among institutions working at improving the GIS capacity of the health sector, as well as between the health sector and other sectors. Improvements should ideally be independent of particular health priorities, as geography has a shared value to all efforts targeting improved health and population outcomes.

The authors of this paper encourage institutions and individuals working or interested in this area, to make themselves known to the Geography of MNH platform in order to strengthen coordination mechanisms and disseminate global public goods.

## Abbreviations

CoIA: Commission on Information and Accountability for Women’s and Children’s Health
GIS: Geographic information systems
iERG: Independent Expert Review Group
MDG: Millennium development goals
MNH: Maternal and newborn health
NSDI: National spatial data infrastructure
SAE: Small area estimation
WHO: World Health Organization

## Footnotes

1 The neonatal period for the newborn baby is normally defined as the first 28 days of life, and the postpartum health of a woman is similarly considered for a month after the birth. However – most of the adverse outcomes related to the birth occur during the first 7 days.

2 The ‘Countdown to 2015’ initiative collects these service data – and Countdown is the official agency for tracking indicators for the Commission on Information and Accountability. Skilled birth attendance – a key MDG indicator– is a crucial human resources indicator [3, 36].

3 For more information: http://integrare.es/mapping-for-maternal-and-newborn-health-innovative-application-of-gis-2/?goback=.gde_4885650_member_229843409.

4 The purpose of this paper is not to compare methods that investigate geographical access to services. Please refer to Higgs [37] or Guagliardo [38] for a comparison and description of several of these methods.
